# Integration of Polypyrrole Electrode into Piezoelectric PVDF Energy Harvester with Improved Adhesion and Over-Oxidation Resistance

**DOI:** 10.3390/polym11061071

**Published:** 2019-06-21

**Authors:** Kyungha Baik, Sohyun Park, Changsang Yun, Chung Hee Park

**Affiliations:** 1 Department of Textiles, Merchandising and Fashion Design, Seoul National University, Seoul 08826, Korea; starlight77@snu.ac.kr; 2 Department of Human Ecology, Korea National Open University, Seoul 03087, Korea; sohyunpark@knou.ac.kr; 3 Department of Fashion Industry, Ewha Womans University, Seoul 03760, Korea

**Keywords:** piezoelectricity, electroconductivity, flexibility, durability, electrode, energy harvester, polypyrrole, poly(vinylidene fluoride)

## Abstract

Smart textiles for wearable devices require flexibility and a lightweight, so in this study, a soft polypyrrole (PPy) electrode system was integrated into a piezoelectric polyvinylidenefluoride (PVDF) energy harvester. The PVDF energy harvester integrated with a PPy electrode had the piezoelectric output voltage of 4.24–4.56 V, while the PVDF energy harvester with an additional aluminum-foil electrode exhibited 2.57 V. Alkaline treatment and chemical vapor deposition with n-dodecyltrimethoxysilane (DTMS) were employed to improve the adhesion between the PVDF and PPy and the resistance to over-oxidation in aqueous solutions. The PVDF film modified by an alkaline treatment could have the improved adhesion via the introduction of polar functional groups to its surface, which was confirmed by the ultrasonication. The surface hydrophobicity of the PPy electrode was enhanced by the DTMS coating, resulting in the improvement of the resistance to over-oxidation with a water contact angle of 111°. Even with the hydrophobic coating, the electrodes remained electroconductive and continued to transfer an electric charge, maintaining the piezoelectricity of the PVDF film. The developed electrode-integrated energy harvester is expected to be applied to smart textiles because it offers the advantages of efficient piezoelectric generation, flexibility, and durability.

## 1. Introduction

Many wearable devices have been made more wearable by incorporating them into garments or accessories, such as watches or bracelets [1,2,3,4,5]. Stretchable, flexible, and lightweight electrical materials and elements are quite valuable for the fabrication of these devices. These applications are of interest to many academic and industrial researchers. Various fiber-based electrical materials and elements have been developed [6,7,8], particularly energy harvesters for wearable devices [9,10,11]. To make wearable devices lighter and more portable, however, improvements to these energy-harvesting devices are required.

Piezoelectric materials become polarized when they undergo strain due to stress, which enables them to convert mechanical energy into electrical energy [12,13]. Repetitive human motions, such as walking or bending at the knees or elbows, can induce energy transformation when applied to piezoelectric energy harvesters [14]. Since they are incorporated into clothing, piezoelectric energy harvesters for wearable devices should be flexible, lightweight, stretchable, biocompatible, and durable. For these reasons, PVDF, a polymeric piezoelectric material, is currently attracting considerable attention [14,15].

However, employing PVDF in energy harvesters can be problematic, because PVDF is not an electroconductive material, so extra electrodes are necessary to transfer an electric charge from the PVDF surface to the circuit [12,13]. Electrode materials must also be flexible and exhibit the same properties that are desired in an energy harvester. PPy, which is an electroconductive polymer, has the advantages of flexibility, biocompatibility, and environmental stability. PPy has also been employed as an electrode material for wearable devices [16,17,18,19]. Despite these advances, there are obstacles preventing the use of PPy as an electrode material for PVDF, such as the weak adhesion force between the PVDF and PPy. PVDF is a fluorocarbon material with a very low surface energy that adheres poorly to other materials [20,21,22,23]. It has also been reported that the adhesion force between the PPy and hydrophobic surfaces is weak [24,25]. This can result in the easy detachment of PPy from PVDF, which reduces the amount of energy that can be transferred from PVDF. Over-oxidation of PPy is another problem that results in the decay of electroconductivity [26,27,28]. Conductivity in electroconductive polymers arises from π-conjugation in their backbone chains [29]. Damage to the pyrrole ring by nucleophiles, such as water or hydroxyl ions, can result in the formation of carbonyl, ester, and hydroxyl groups, or irreversible ring opening. As a result of the nucleophilic attack, the π-conjugation was reduced and the electrical conductivity eventually decreased [26,30,31,32,33,34,35].

Several methods have been used to modify PVDF surfaces, including alkaline treatment [36], plasma treatment [20,21], ion-beam irradiation [22,23], and graft copolymerization [22,37]. Brewis et al. [38] and Ross et al. [39] proposed mechanisms for explaining the structural changes in PVDF during alkaline treatment. The PVDF surface became more hydrophilic by alkaline treatment through the introduction of C=C double bonds, carbonyl groups, and hydroxyl groups to the polymer chain.

Some researchers have attempted to improve the adhesion force of PPy on substrates. Liu et al. [40] reported that PPy could interact with a substrate not only through the van der Waals forces, but also via the participation of its N–H groups in hydrogen bonding. According to Nickels et al. [41] and Romero et al. [42], a negative surface charge on the substrate and the delocalized positive charge on the PPy chain can be involved in the electrostatic interaction.

This study aimed to develop a flexible, electroconductive piezoelectric device that could have good resistance to over-oxidation and improved adhesion between the piezoelectric PVDF and electroconductive PPy. To enhance its adhesive properties, PVDF was subjected to alkaline treatment prior to PPy polymerization. Hydrophobic coating by DTMS chemical vapor deposition (CVD) was also performed to protect the PPy electrode from reduction by water.

## 2. Materials and Methods

### 2.1. Materials

PVDF film (thickness; 0.08 mm) was purchased from Fils Co., Ltd. (Seongnam, Republic of Korea), and was ultrasonicated in water and washed with ethanol prior to use. Anhydrous ethanol (99.9%, Daejung, Siheung, Republic of Korea), sodium hydroxide (guaranteed reagent, JUNSEI, Tokyo, Japan), pyrrole (reagent grade; 98%, Sigma Aldrich, St. Louis, MO, USA), anthraquinone-2-sulfonic acid sodium salt monohydrate (AQSA-Na, 97%, Sigma Aldrich, St. Louis, MO, USA), FeCl_3_·6H_2_O (reagent grade; ≥98%, Sigma Aldrich, St. Louis, MO, USA), and DTMS (>93%, Tokyo Chemical Industry Co., Ltd., Tokyo, Japan) were used as received without any further purification.

### 2.2. Fabrications

#### 2.2.1. Alkaline Treatment

PVDF films were immersed in 6 M aqueous NaOH at 60 °C for 60, 120, and 180 min. After treatment, they were washed with distilled water, rinsed with ethanol, and then dried at room temperature.

#### 2.2.2. Polypyrrole Polymerization for Electrode Formation

Electrodes were obtained through the polymerization of PPy on the PVDF film surfaces according to a previously reported method [43,44,45]. Pyrrole (0.69 mL) and AQSA-Na (0.41 g, 1.25 mmol) were dissolved in 50 mL of distilled water to yield solution A. FeCl_3_·6H_2_O (2.7 g, 0.01 mol) was dissolved in 50 mL of distilled water to yield solution B. Solutions A and B were cooled in a bath for 1 h with stirring and maintained at 5 °C until the polymerization step. PVDF film was immersed in solution A for 20 min with stirring. Solution B was then added, drop-by-drop, to solution A over 20 min. PPy polymerization was done for a total of 1 h. After PPy polymerization, the films were treated by rinsing, ultrasonication, and re-rinsing with ethanol. The samples were then dried at room temperature.

#### 2.2.3. Hydrophobic Coating by Chemical Vapor Deposition

A hydrophobic coating of DTMS was applied to a film via CVD. Each film was placed in a WL1900 oven (With Lab Co. Ltd., Gunpo, Republic of Korea) in the condition with 600 µL of DTMS. CVD was performed for 3 h at 80 °C. The films were then dried for 1 h. This process was repeated three times.

**Figure 1:**
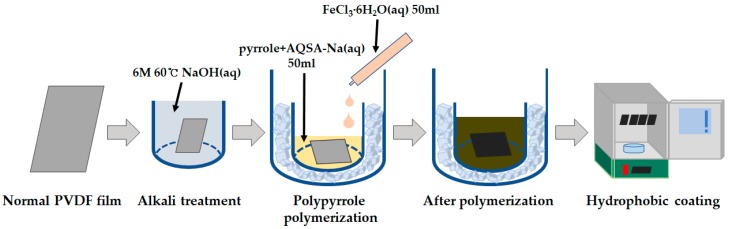
Illustration of experiment procedure for polyvinylidenefluoride (PVDF) film.

The fabrication procedure is shown in Figure 1, and the codes used to identify samples are summarized in Table 1.

### 2.3. Characterization

#### 2.3.1. Morphology

The morphologies of the PVDF films were analyzed by field-emission scanning electron microscopy using an AURIGA Series FE-SEM (Carl Zeiss, Oberkochen, Germany).

#### 2.3.2. Surface Resistivity

According to the AATCC 76-1995 Test Method, the surface resistivity of the PVDF films was measured with a GOM-804 DC milliohm meter (GW INSTEK, Taipei, Taiwan) [46]. Each film was cut into 40 mm × 62 mm and placed over copper strips. The surface resistivity of each sample was calculated using the Equation (1).

(1) $$R_{S}=R_{M}\times \frac{w}{d}$$

$R_{S}$: Surface resistivity of sample;
$R_{M}$: Resistance measured with a DC milliohm meter;
w: Width of copper strip (30 mm);
d: Distance between copper strips (20 mm).

#### 2.3.3. Water Contact Angle

The water contact angle was measured by a Theta Lite Optical Tensiometer (KSV Instruments, Helsinki, Finland) to evaluate the wettability of the samples.

#### 2.3.4. Fourier-Transform Infrared (FT-IR) Spectroscopy

In order to examine the functional groups introduced on the surface of PVDF film by the alkaline treatment, FT-IR spectra were recorded using a FT-IR spectrophotometer Nicolet 6700 (Thermo Scientific, Madison, WI, USA) with an ATR accessory. The spectra were acquired at 8 cm^−1^ resolution and 32 scans with a wavenumber range of 650–4000 cm^−1^.

#### 2.3.5. Piezoelectricity

The piezoelectric output voltage and current were measured to evaluate the piezoelectric properties of the samples. The piezoelectric output voltage was measured with a 2182 A nanovoltmeter (Keithley, Cleveland, OH, USA), and the piezoelectric output current was measured with a high-power electrochemical analyzer (IviumStat.h, Ivium Technologies, Eindhoven, The Netherlands). Samples were cut into 40 mm × 25 mm. For the PVDF films without PPy electrodes, aluminum foil was attached to both sides to serve as electrodes. The PVDF films that underwent PPy polymerization did not require aluminum foil, since the PPy functioned as the electrode. The piezoelectric output voltage and current were measured for 60 s while the sample was subjected to regular bending movements. The bending movements were performed manually until both sides of a film were matched in half, and their bending and releasing cycle was repeated with a frequency of 1 Hz [47].

#### 2.3.6. Adhesion Durability

To evaluate the changes in the adhesion strength enhanced by the alkaline treatment, the films were ultrasonicated. Each sample was immersed in 50 mL of anhydrous ethanol and ultrasonicated [48,49,50]. After each treatment, the surface resistivity was measured.

#### 2.3.7. Resistance to Over-Oxidation by Water

The samples were immersed in 50 mL of distilled water for 5 min to evaluate their resistance to over-oxidation. The samples were then dried at room temperature for 90 min, and the surface resistivity was measured. This cycle was repeated three times with each sample. The change in the surface resistivity was calculated using Equation (2).

(2) $$RC\;\left(\%\right)=\frac{R_{s}-R_{s_{0}}}{R_{s_{0}}}\times 100.$$

RC: Change in sample surface resistivity;
$R_{s_{0}}$: Surface resistivity of non-immersed sample;
$R_{s}$: Surface resistivity of sample after immersion.

## 3. Results and Discussion

### 3.1. Enhancement of Adhesive Property of PVDF Film by Alkaline Treatment

**Figure 2:**
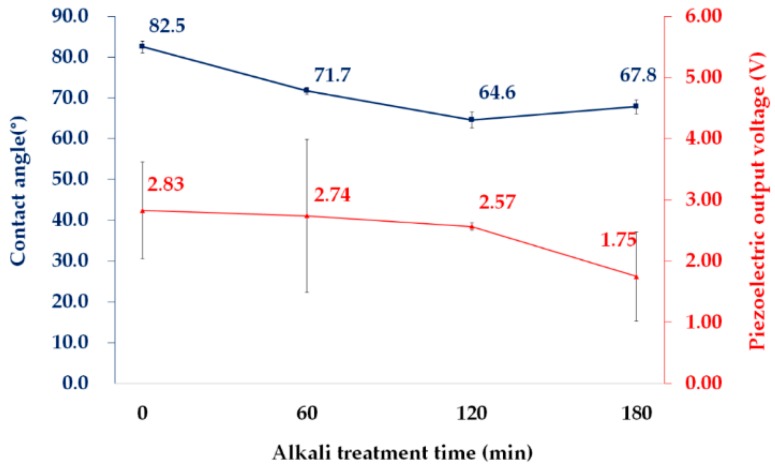
Water contact angle and piezoelectric output voltage of the PVDF film treated with an NaOH solution.

**Figure A1:**
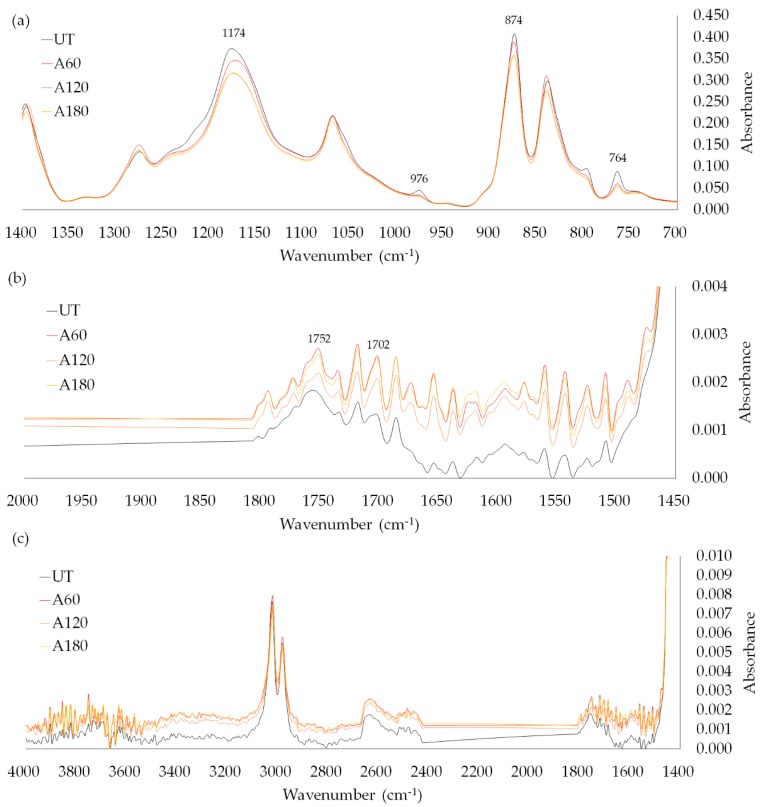
FT-IR spectra of PVDF films treated with an NaOH solution for 0 min (UT), 60 min (A60), 120 min (A120), and 180 min (A180) for the range of 700–1400 cm^−1^ (**a**), 1450–2000 cm^−1^ (**b**), 1400–4000 cm^−1^ (**c**).

To enhance the adhesion of electroconductive PPy to PVDF film, an alkaline treatment was carried out for a range of durations. The results are shown in Figure 2 with respect to the water contact angles and the piezoelectric effect of the PVDF film. The PVDF surface became more hydrophilic by alkaline treatment through the introduction of C=C double bonds (1752 cm^−1^ and 1702 cm^−1^) and hydroxyl groups (2800–3600 cm^−1^), which was confirmed by the FT-IR results in Figure A1 [51,52]. The most noticeable decrease in the water contact angle, from 82.5° to 64.6°, was observed at 120 min of alkaline treatment, indicating an increase in the adhesive property of the PVDF film. When a PVDF film was treated for 180 min, the water contact angle rebounded by the increasing of elimination of HF [36,39]. Although the piezoelectric output voltage decreased with an increase in the alkaline treatment time, the decrease was unremarkable until 120 min. Based on these results, the alkaline treatment was determined with 120 min in the subsequent experiments.

### 3.2. Effects of Alkaline Treatment, PPy Polymerization, and Hydrophobic Coating on Properties of PVDF Films

#### 3.2.1. Morphology

**Figure 3:**
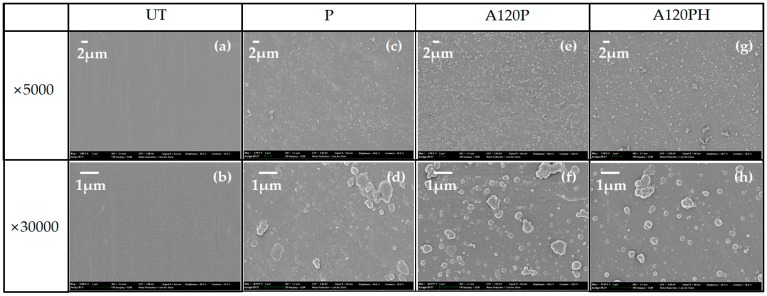
FE-SEM images for untreated sample (UT) (**a**,**b**), polypyrrole coated PVDF film without alkaline treatment (P; **c**,**d**), polypyrrole coated PVDF film after alkaline treatment for 120 min (A120P; **e**,**f**), and hydrophobic coated PVDF film after alkaline treatment for 120 min and polypyrrole coating (A120PH; **g**,**h**).

The PVDF film surfaces treated in an NaOH solution and followed by PPy polymerization and DTMS chemical deposition are shown in Figure 3. The untreated sample shown in Figure 3a,b had a smooth surface. While sample PPy coated PVDF film without alkaline treatment (P) exhibited a nearly flat and continuous structure (Figure 3c,d), the surface of PPy coated PVDF film after alkaline treatment for 120 min (A120P) in Figure 3e,f was covered with hundreds of nanometer-scale particles, which were connected by a continuous matte structure. The PPy structure of a hydrophobic-coated PVDF film after alkaline treatment for 120 min and PPy coating (A120PH) in Figure 3g,h was similar to that of A120P, confirming that the hydrophobic coating by chemical deposition did not affect the surface morphology.

According to Hwang et al. [53], the growth mechanism of an electroconductive polymer is similar to that of metals. The growth may proceed either by instantaneous nucleation and two-dimensional growth (instantaneous 2D), or by progressive nucleation and three-dimensional growth (progressive 3D). In the case of two-dimensional growth, most of the monomers adsorb onto the substrate to form oligomers, making a dense monolayer on the substrate surface. In three-dimensional growth, however, oligomers are produced in the bulk solution, not on the substrate. Then, the oligomers precipitate on the substrate surface and grow in three dimensions. Since the solubility of pyrrole monomers in water is limited, monomers in aqueous solutions prefer to adsorb onto hydrophobic substrates. High concentrations of pyrrole radical cations and pyrrole monomers on hydrophobic surface result in continuous PPy growth. These pyrrole monomers cover hydrophobic substrates quickly, and their polymerization leads to the formation of a continuous granular matte structure (instantaneous 2D). In contrast, relatively few pyrrole monomers adsorb to hydrophilic substrates and grow at fewer sites. This results in the discontinuous growth of PPy to form discrete spherical nanoparticle structures (progressive 3D) [24,54,55,56].

**Figure 4:**
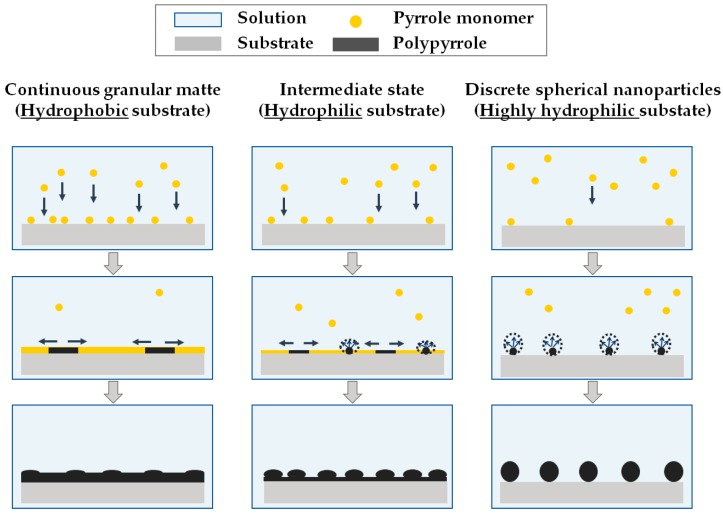
Diagram of nucleation and growth mechanism and morphology of polypyrrole according to the hydrophilicity of substrates.

Sample P in Figure 3c,d exhibited a continuous and granular matte structure due to the hydrophobic surface of the PVDF film. However, nanoparticles were more visible on the surface of the A120P than on that of sample P, and they were still connected by a matte structure, between that of discrete spherical nanoparticles and a continuous granular matte structure [24,55,57,58]. This was possibly caused by the difference in the hydrophilicity of the untreated sample (UT) and A120 substrates. Since UT is hydrophobic, pyrrole was polymerized on its surface in 2D, making the surface of sample P continuous and granular matte. However, A120 with a more hydrophilic substrate surface and sparse pyrrole coating caused the A120P surface to fall between 2D and 3D, assuming an intermediate structure between a continuous granular matte and a discrete spherical nanoparticle structure, a so-called “semi-discrete” structure. Schematic diagrams of the continuous granular matte, semi-discrete, and discrete spherical nanoparticles are shown in Figure 4.

#### 3.2.2. Wettability

**Figure 5:**
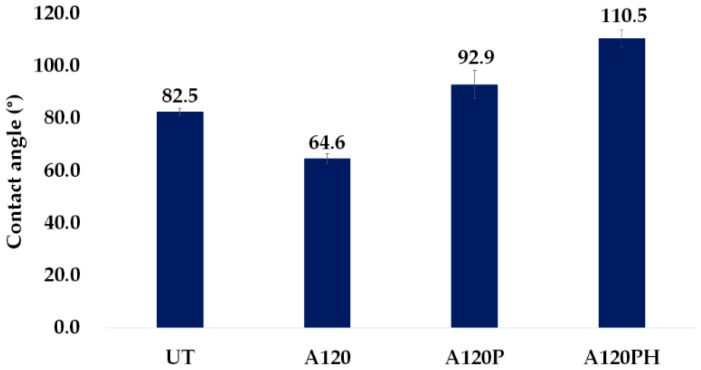
Water contact angle of an untreated sample (UT), a sample treated with an NaOH solution (A120), a sample treated with an NaOH solution and followed by PPy polymerization (A120P), and a sample treated with an NaOH solution and followed by PPy polymerization and hydrophobic coating (A120PH).

The wettability of the samples was evaluated from the water contact angles, shown in Figure 5. The water contact angle on the alkaline-treated PVDF film (A120) was 64.6°, which was attributed to hydrophilic groups introduced during alkaline treatment. Following PPy polymerization (A120P), the contact angle increased to 92.9°. After coating the film with DTMS by CVD (A120PH), the contact angle increased from 92.9° to 110.5°. It was thought that A120PH would have the highest water contact angle due to the introduction of nanostructures by polymerization and the surface energy lowered by DTMS coating. However, this was a lower value than was reported previously for a hydrophobic DTMS coating on the fabric [45,59]. Fabrics are comprised of yarns, which provide intrinsic microscale roughness, and additional nanostructures are very effective for creating a hierarchical roughness to ensure the superhydrophobicity and the minimum contact area with water [59]. In this study, PPy was polymerized on a film substrate with a smooth surface to yield only a semi-discrete nanostructure. Therefore, even after the application of a DTMS coating, the water contact angle did not increase greatly.

#### 3.2.3. Electric and Piezoelectric Properties

**Figure 6:**
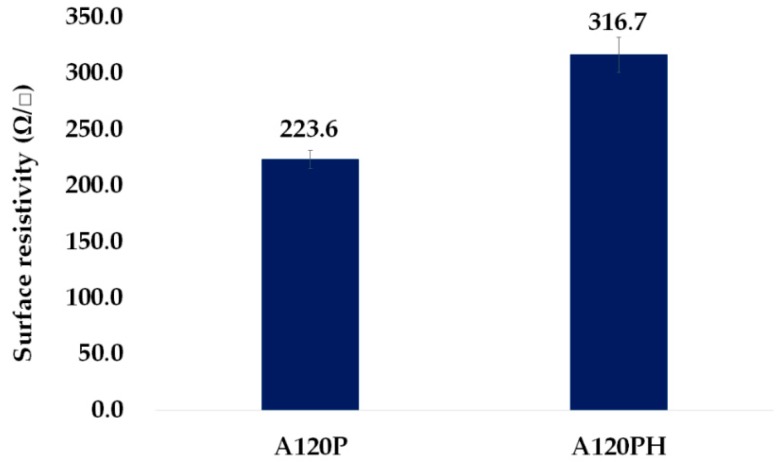
Surface resistivity of a sample treated with an NaOH solution and followed by PPy polymerization (A120P), and a sample treated with an NaOH solution and followed by PPy polymerization and hydrophobic coating (A120PH).

As shown in Figure 6, the surface resistivity of A120P was 223.6 Ω/sq and that of A120PH was 316.7 Ω/sq. The introduction of intrinsically conducting polymer to the surface made the non-conductive PVDF film electro-conductive. It was reported that the electroconductivity was significantly low in the discrete spherical nanoparticle structure, which was formed by polymerization on a hydrophilic surface [55]. The poor electroconductivity of this structure indicated that it was not suitable for use as an electrode. However, the morphological continuity of PPy formed a semi-discrete structure by polymerization on a less hydrophilic surface, and it exhibited electroconductivity, indicating that it could be used as an electrode.

The surface resistivity of A120PH was 42% higher than that of A120P, because the PPy introduced to the surface by polymerization was covered with a hydrophobic coating, which interfered with the flow of electrons. Even so, the A120PH was sufficiently electro-conductive to allow its use as an electrode, because its surface resistivity was much lower than the criterion of 1.0 × 10^5^ Ω/sq for conductive materials in the Electronic Industries Association (EIA) standard [60].

**Figure 7:**
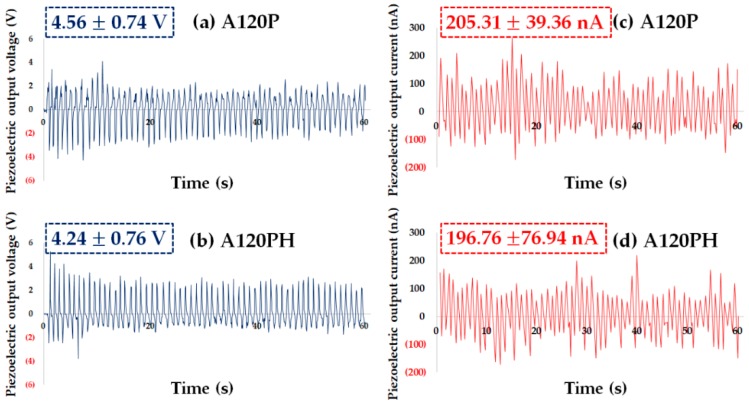
Piezoelectric output voltage of (**a**) a sample treated with an NaOH solution and followed by PPy polymerization (A120P) and (**b**) a sample treated with an NaOH solution and followed by PPy polymerization and hydrophobic coating (A120PH) and piezoelectric output current of (**c**) A120P and (**d**) A120PH (values on the upper left of each graph are average values of six samples in the peak to peak values).

The piezoelectric output voltage and current from the A120P and A120PH samples are shown in Figure 7. While PVDF films (UT, A60, A120, A180) with aluminum-foil electrodes exhibited 1.75–2.83 V for their piezoelectric output voltage, as shown in Figure 2, the A120P and A120PH exhibited piezoelectric output voltages of 4.56 and 4.24 V, respectively. When the electrode was not integrated into an energy harvester, deformation might occur if force were applied between the electrode and the energy harvester, causing the output voltage to decrease. Therefore, the A120P and A120PH may exhibit higher output voltages and currents than the films with an additional electrode. The output voltage and current of A120PH were lower than those of A120P by 6.92% and 4.17%, respectively. The difference in the piezoelectric property may be smaller than the difference in the electrical conductivity. It was because that the generated current flowed mainly along the electroconductive PPy layer without interfering with the hydrophobic coating layer, and then delivered to the measuring device. These results confirmed that the PPy electrode performed normally, as expected, and that the hydrophobic coating did not have a significant impact on the piezoelectric properties of the films.

### 3.3. Durability

#### 3.3.1. Adhesion between PVDF Films and PPy Electrodes

**Figure 8:**
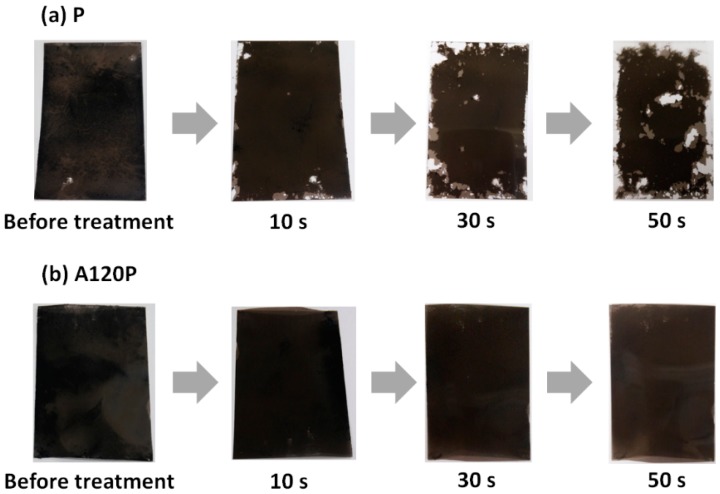
Removal of PPy layer in (**a**) a sample covered by only PPy polymerization (P) and (**b**) a sample treated with an NaOH solution and followed by PPy polymerization (A120P) according to the duration of the ultrasonication.

**Figure A2:**
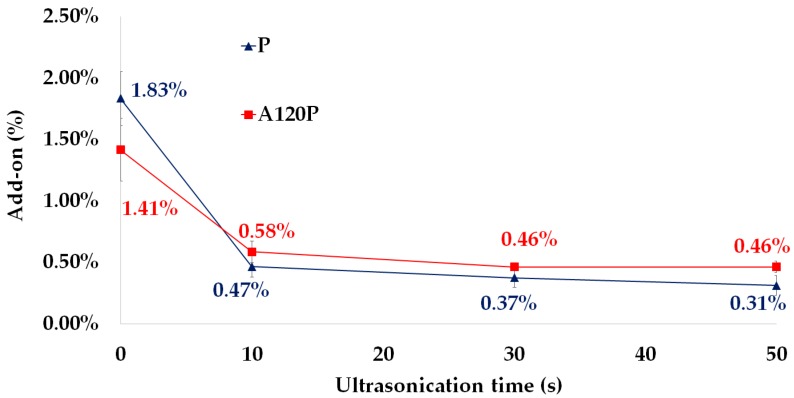
Changes in the add-on (%) of a sample covered by only PPy polymerization (P) and a sample treated with an NaOH solution and followed by PPy polymerization (A120P) according to the ultrasonication duration.

To verify that the alkaline treatment improved adhesion and durability, samples A120P and P were washed by the ultrasonication. Figure 8 shows the changes in the PPy electrodes of samples P and A120P with an increase in the duration of the ultrasonication treatment. The PPy layer on sample P was gradually removed as the duration of the ultrasonication increased. In contrast, the PPy layer on the A120P remained intact, even after treatment for 50 s. The maximum time was set to 50 s because there was no significant change in weight after 30 s, as shown in Figure A2.

Since the surface of the untreated PVDF film lacked hydroxyl and carbonyl groups that could interact with the PPy, an interaction occurred only as a result of the van der Waals forces, so that the adhesion between the PVDF and PPy on sample P was weak. On the other hand, because of the hydrophilic groups introduced onto the surface of the A120P by the alkaline treatment, hydrogen bonding and electrostatic interaction between the negative and positive charges was possible [40,41], and this led to the improved durability of adhesion for the A120P sample.

**Figure 9:**
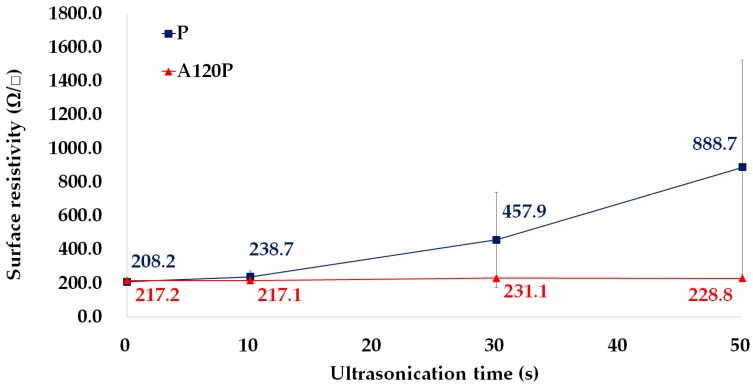
Change of the surface resistivity of a sample covered by only PPy polymerization (P) and a sample treated with an NaOH solution and followed by PPy polymerization (A120P) according to the ultrasonication duration.

Figure 9 shows that the adhesive property of the PPy electrode was improved by the alkaline treatment in terms of the surface resistivity. After treatment for 50 s, the surface resistivity of sample P was four times larger than its initial state. However, the surface resistivity of the A120P was nearly unchanged. The granular matte surface of sample P appeared to be damaged, which removed or reduced the paths for the flow of electrons. Thus, the movement of the electrons was impeded, and the surface resistivity of the sample increased. The PPy layer of the A120P remained intact, and its surface resistivity was almost unchanged.

#### 3.3.2. Resistance to Over-Oxidation by Water

**Figure 10:**
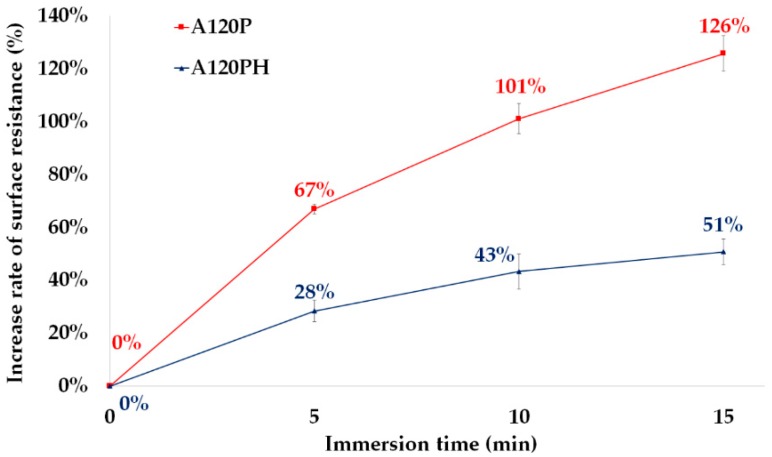
Surface resistivity of a sample treated with an NaOH solution and followed by PPy polymerization (A120P) and a sample treated with an NaOH solution and followed by PPy polymerization and hydrophobic coating (A120PH) by the duration of the immersion at water.

As the duration of the water immersion increased, the surface resistivity of both the A120P and A120PH also increased (Figure 10). It was thought that the over-oxidation by water immersion brought the loss of charge, which lead to the increase in the surface resistivity [61,62]. After immersion for 15 min, the surface resistivity of the A120P increased by 126%, but that of A120PH increased by only 51%. The surface resistivity of the A120P increased at a rate that was more than twice that of A120PH at every stage. It appeared that the hydrophobic coating formed a physical barrier on the PPy layer by preventing the contact between the PPy and the water. For this reason, the resistance to over-oxidation of PPy in the A120PH was better than that of PPy in the A120P.

## 4. Conclusions

In this study, PPy electrodes were combined with PVDF films for use in a flexible and practical piezoelectric energy harvester. The PVDF energy harvester integrated with PPy electrode had the piezoelectric output voltage of 4.24–4.56 V, while the PVDF energy harvester with an additional aluminum-foil electrode exhibited 2.57 V. The adhesion between the PVDF and PPy was modified by alkaline treatment, and enhancements in the resistance to over-oxidation by the application of a hydrophobic DTMS coating were evaluated. The morphology of PPy without alkaline treatment was a continuous granular matte structure, while a semi-discrete structure was observed on the PVDF films treated with the alkaline solution. Improved adhesion between the alkaline-treated PVDF and PPy was verified through ultrasonication tests. The PPy electrode polymerized on the alkaline-treated PVDF was not removed or damaged, and the surface resistivity was increased by 5.34%. Although the surface resistivity of the PPy electrode became higher with a hydrophobic coating, its piezoelectric output voltage and current were reduced by 6.92% and 4.17%, respectively. The electron transfer was not adversely affected by the increased hydrophobicity, while the hydrophobic coating enhanced the over-oxidation resistance of the PPy electrode.

Alkaline treatment and the DTMS coating via CVD reinforced the adhesion between PVDF and PPy and improved the resistance to over-oxidation of PPy electrodes. Furthermore, the low cost of PPy, its simple polymerization process, and the potential for large-scale manufacture make PPy a promising electrode material. Since the PPy electrode was applied to PVDF films without any adhesive paste, their flexible and stretchable properties were not significantly affected. The feasibility of PVDF as an energy harvester in wearable devices was reinforced by the strong connection between the PPy electrode and the PVDF film. More precise control of the PPy structure is suggested to achieve the superhydrophobic or self-cleaning properties, and such improvements would prevent degradation of the electrode upon exposure to rain or pollutants. Based on the results of this research, we expect further development of the flexible and stretchable properties of piezoelectric energy harvesters, weight-reduction, and advances in wearable device technology.

## Figures and Tables

**Table 1:** Sample code and treatment description.

Samples	Alkaline Treatment (Duration; min)	Polypyrrole Polymerization	Hydrophobic Coating
UT	×	×	×
P	×	O	×
A120	O (120)	×	×
A120P	O (120)	O	×
A120PH	O (120)	O	O

## References

[1] Crawford K., Lingel J., Karppi T. (2015). Our metrics, ourselves: A hundred years of self-tracking from the weight scale to the wrist wearable device. Eur. J. Cult. Stud..

[2] Do Q., Martini B., Choo K.K.R. (2017). Is the data on your wearable device secure? An Android Wear smartwatch case study. Softw. Pract. Exp..

[3] Kroll R.R., Boyd J.G., Maslove D.M. (2016). Accuracy of a wrist-worn wearable device for monitoring heart rates in hospital inpatients: A prospective observational study. J. Med. Internet Res..

[4] Koo S.H., Fallon K. (2018). Explorations of wearable technology for tracking self and others. Fash. Text..

[5] Lee Y.A., Koo S.H. (2018). Introduction to special collection on 3D printing and wearable technology in fashion. Fash. Text..

[6] Matsuhisa N., Kaltenbrunner M., Yokota T., Jinno H., Kuribara K., Sekitani T., Someya T. (2015). Printable elastic conductors with a high conductivity for electronic textile applications. Nat. Commun..

[7] Shyr T.W., Shie J.W., Jiang C.H., Li J.J. (2014). A textile-based wearable sensing device designed for monitoring the flexion angle of elbow and knee movements. Sensors.

[8] Cherenack K., Zysset C., Kinkeldei T., Münzenrieder N., Tröster G. (2010). Woven electronic fibers with sensing and display functions for smart textiles. Adv. Mater. (Weinh. Ger.).

[9] Seung W., Gupta M.K., Lee K.Y., Shin K.S., Lee J.H., Kim T.Y., Kim S., Lin J., Kim J.H., Kim S.W. (2015). Nanopatterned textile-based wearable triboelectric nanogenerator. ACS Nano.

[10] Bhattacharya R., de Kok M.M., Zhou J. (2009). Rechargeable electronic textile battery. Appl. Phys. Lett..

[11] Soin N., Shah T.H., Anand S.C., Geng J., Pornwannachai W., Mandal P., Reid D., Sharma S., Hadimani R.L., Bayramol D.V. (2014). Novel “3-D spacer” all fibre piezoelectric textiles for energy harvesting applications. Energy Environ. Sci..

[12] Ramadan K.S., Sameoto D., Evoy S. (2014). A review of piezoelectric polymers as functional materials for electromechanical transducers. Smart Mater. Struct..

[13] Park T., Kim B., Kim Y., Kim E. (2014). Highly conductive PEDOT electrodes for harvesting dynamic energy through piezoelectric conversion. J. Mater. Chem. A Mater..

[14] Fuh Y.K., Chen P.C., Huang Z.M., Ho H.C. (2015). Self-powered sensing elements based on direct-write, highly flexible piezoelectric polymeric nano/microfibers. Nano Energy.

[15] Proto A., Vlach K., Conforto S., Kasik V., Bibbo D., Vala D., Bernabucci I., Penhaker M., Schmid M. (2017). Using PVDF films as flexible piezoelectric generators for biomechanical energy harvesting. Lékař Technika Clin. Technol..

[16] Mykhailiv O., Imierska M., Petelczyc M., Echegoyen L., Plonska-Brzezinska M.E. (2015). Chemical versus Electrochemical Synthesis of Carbon Nano-onion/Polypyrrole Composites for Supercapacitor Electrodes. Chem. Eur. J..

[17] Huang Y., Li H., Wang Z., Zhu M., Pei Z., Xue Q., Huang Y., Zhi C. (2016). Nanostructured Polypyrrole as a flexible electrode material of supercapacitor. Nano Energy.

[18] Hebeish A., Farag S., Sharaf S., Shaheen T.I. (2016). Advancement in conductive cotton fabrics through in situ polymerization of polypyrrole-nanocellulose composite. Carbohydr. Polym..

[19] Li Y., Neoh K.G., Kang E.T. (2004). Plasma protein adsorption and thrombus formation on surface functionalized polypyrrole with and without electrical stimulation. J. Colloid Interface Sci..

[20] Zheng Z., Gu Z., Huo R., Ye Y. (2009). Superhydrophobicity of polyvinylidene fluoride membrane fabricated by chemical vapor deposition from solution. Appl. Surf. Sci..

[21] Park Y.W., Inagaki N. (2003). Surface modification of poly(vinylidene fluoride) film by remote Ar, H_2_, and O_2_ plasmas. Polymer.

[22] Lee J.S., Kim G.H., Hong S.M. (2008). Effect of complex Ion beam/plasma treatment of the surface functionalization and crystal phase transition of piezoelectric poly(vinylidene fluoride). Mol. Cryst. Liq. Cryst..

[23] Han S., Choi W.K., Yoon K.H., Koh S.K. (1999). Surface reaction on polyvinylidenefluoride (PVDF) irradiated by low energy ion beam in reactive gas environment. J. Appl. Polym. Sci..

[24] Huang Z., Wang P.C., MacDiarmid A.G., Xia Y., Whitesides G. (1997). Selective deposition of conducting polymers on hydroxyl-terminated surfaces with printed monolayers of alkylsiloxanes as templates. Langmuir.

[25] Huang Z., Wang P.C., Feng J., MacDiarmid A.G., Xia Y., Whitesides G.M. (1997). Selective deposition of films of polypyrrole, polyaniline and nickel on hydrophobic/hydrophilic patterned surfaces and applications. Synth. Met..

[26] Thieblemont J.C., Brun A., Marty J., Planche M.F., Calo P. (1995). Thermal analysis of polypyrrole oxidation in air. Polymer.

[27] Sixou B., Mermilliod N., Travers J.P. (1996). Aging effects on the transport properties in conducting polymer polypyrrole. Phys. Rev. B Condens. Matter Mater. Phys..

[28] Ibanez J.G., Alatorre-Ordaz A., Gutierrez-Granados S., Batina N. (2008). Nanoscale degradation of polypyrrole films under oxidative stress: An atomic force microscopy study and review. Polym. Degrad. Stab..

[29] Balint R., Cassidy N.J., Cartmell S.H. (2014). Conductive polymers: Towards a smart biomaterial for tissue engineering. Acta Biomater..

[30] Liu Y.C., Hwang B.J. (2001). Mechanism of conductivity decay of polypyrrole exposed to water and enhancement of conductivity stability of copper (I)-modified polypyrrole. J. Electroanal. Chem..

[31] Beck F., Braun P., Oberst M. (1987). Organic Electrochemistry in the Solid State-Overoxidation of Polypyrrole. Ber. Bunsenges. Physik. Chem..

[32] Pyo M., Reynolds J.R., Warren L.F., Marcy H.O. (1994). Long-term redox switching stability of polypyrrole. Synth. Met..

[33] Otero T.F., Tejada R., Elola A.S. (1987). Formation and modification of polypyrrole films on platinum electrodes by cyclic voltammetry and anodic polarization. Polymer.

[34] Mattila H.R. (2006). Intelligent Textiles and Clothing.

[35] Münstedt H. (1988). Ageing of electrically conducting organic materials. Polymer.

[36] Zheng Z., Gu Z., Huo R., Luo Z. (2010). Superhydrophobic poly(vinylidene fluoride) film fabricated by alkali treatment enhancing chemical bath deposition. Appl. Surf. Sci..

[37] Liu Y.X., Kang E.T., Neoh K.G., Tan K.L. (2000). Surface modification of poly(vinylidene fluoride) films by graft copolymerization for adhesion improvement with evaporated metals. J. Macromol. Sci. Part A Pure Appl. Chem..

[38] Brewis D.M., Mathieson I., Sutherland I., Cayless R.A., Dahm R.H. (1996). Pretreatment of poly(vinyl fluoride) and poly(vinylidene fluoride) with potassium hydroxide. Int. J. Adhes. Adhes..

[39] Ross G.J., Watts J.F., Hill M.P., Morrissey P. (2000). Surface modification of poly(vinylidene fluoride) by alkaline treatment1. The degradation mechanism. Polymer.

[40] Liu Y., Zhao X., Tuo X. (2017). Preparation of polypyrrole coated cotton conductive fabrics. J. Text. Inst..

[41] Nickels J.D., Schmidt C.E. (2013). Surface modification of polypyrrole via affinity peptide: Quantification and mechanism. J. Mater. Chem. B.

[42] Romero I.S., Schurr M.L., Lally J.V., Kotlik M.Z., Murphy A.R. (2013). Enhancing the interface in silk–polypyrrole composites through chemical modification of silk fibroin. ACS Appl. Mater. Interfaces.

[43] Huang Y.M., Zhou F.F., Deng Y., Zhai B.G. (2008). Effects of salt 9, 10-anthraquinone-2-sulfonic acid sodium on the conductivity of polypyrrole. Solid State Ion..

[44] Huang G., Liu L., Wang R., Zhang J., Sun X., Peng H. (2016). Smart color-changing textile with high contrast based on a single-sided conductive fabric. J. Mater. Chem. C Mater..

[45] Lee S., Park C.H. (2018). Electric heated cotton fabrics with durable conductivity and self-cleaning properties. RSC Adv..

[46] American Association of Textile Chemists and Colorists (1996). Electrical Resistivity of Fabrics; AATCC Test Method 79-1995. Technical Manual of AATCC.

[47] Ju B.J., Oh J.H., Yun C., Park C.H. (2018). Development of a superhydrophobic electrospun poly(vinylidene fluoride) web via plasma etching and water immersion for energy harvesting applications. RSC Adv..

[48] Moses S., Witt R.K. (1949). Evaluation of adhesion by ultrasonic vibrations. Ind. Eng. Chem..

[49] Moses S. (1949). The nature of adhesion. Ind. Eng. Chem..

[50] Mittal K.L. (1976). Adhesion measurement of thin films. Act. Passiv. Electron. Compon..

[51] Zhang S., Shen J., Qiu X., Weng D., Zhu W. (2006). ESR and vibrational spectroscopy study on poly(vinylidene fluoride) membranes with alkaline treatment. J. Power Sour..

[52] Grasselli M., Betz N. (2005). Making porous membranes by chemical etching of heavy-ion tracks in β-PVDF films. Nucl. Instr. Meth. Phys. Res. B.

[53] Hwang B.J., Santhanam R., Lin Y.L. (2000). Nucleation and growth mechanism of electropolymerization of polypyrrole on gold/highly oriented pyrolytic graphite electrode. J. Electrochem. Soc..

[54] Goren M., Qi Z., Lennox R.B. (2000). Selective templated growth of polypyrrole strands on lipid tubule edges. Chem. Mater..

[55] Wang P.C., Lakis R.E., MacDiarmid A.G. (2008). Morphology-correlated electrical conduction in micro-contact-printed polypyrrole thin films grown by in situ deposition. Thin Solid Films.

[56] Wang P.C., Huang Z., MacDiarmid A.G. (1999). Critical dependency of the conductivity of polypyrrole and polyaniline films on the hydrophobicity/hydrophilicity of the substrate surface. Synth. Met..

[57] Huang J., Kim F., Tao A.R., Connor S., Yang P. (2005). Spontaneous formation of nanoparticle stripe patterns through dewetting. Nat. Mater..

[58] Bhattacharya S., Datta A., Berg J.M., Gangopadhyay S. (2005). Studies on surface wettability of poly(dimethyl) siloxane (PDMS) and glass under oxygen-plasma treatment and correlation with bond strength. J. Microelectromech. Syst..

[59] Park Y., Park C.H., Kim J. (2014). A quantitative analysis on the surface roughness and the level of hydrophobicity for superhydrophobic ZnO nanorods grown textiles. Text. Res. J..

[60] Singh S.P., El-Khateeb H. (1994). Evaluation of a proposed test method to measure surface and volume resistance of static dissipative packaging materials. Packag. Technol. Sci..

[61] Sabbatini L., Malitesta C., Giglio E.D., Losito I., Torsi L., Zambonin P.G. (1999). Electrosynthesised thin polymer films: The role of XPS in the design of application oriented innovative materials. J. Electron Spectrosc. Relat. Phenom..

[62] Liu Y.C. (2004). Characteristics of vibration modes of polypyrrole on surface-enhanced Raman scattering spectra. J. Electroanal. Chem..

